# Anti-*Candida*, Anti-Enzyme Activity and Cytotoxicity of 3,5-Diaryl-4,5-dihydro-1*H*-pyrazole-1-carboximidamides

**DOI:** 10.3390/molecules19055806

**Published:** 2014-05-06

**Authors:** Simone Oliveira, Lucas Pizzuti, Frank Quina, Alex Flores, Rafael Lund, Claiton Lencina, Bruna S. Pacheco, Claudio M. P. de Pereira, Evandro Piva

**Affiliations:** 1 Laboratory of Microbiology, Postgraduate Program in Dentistry, School of Dentistry, Federal University of Pelotas (UFPel)—Rua Gonçalves Chaves, 457/504, Pelotas, RS 96015-000, Brazil; E-Mail: sisi_mone@hotmail.com; 2 Faculty of Exact Sciences and Technology, Federal University of Grande Dourados, Dourados, MS 79825-070, Brazil; E-Mail: lucas.pizzuti@gmail.com; 3 Department of Chemistry and the Consortium for Photochemical Technology (NAP-PhotoTech), University of São Paulo, São Paulo, SP 05508-000, Brazil; E-Mail: quina@usp.br; 4 School of Chemistry and Food, Federal University of Rio Grande, Rio Grande, RS 95500-000, Brazil; E-Mail: alex@pesquisador.cnpq.br; 5 Laboratory of Bioactive Heterocycles and Bioprospection (LAHBBio), Center for Chemical, Pharmaceutical and Food Sciences, Federal University of Pelotas, Pelotas, RS 96010-610, Brazil; E-Mails: leonetti.lencina@gmail.com (C.L.); bruna.spacheco@hotmail.com (B.S.P.); claudiochemistry@gmail.com (C.M.P.P.); 6 Department of Restorative Dentistry, School of Dentistry, Federal University of Pelotas (UFPel), Pelotas, RS 96015-000, Brazil; E-Mail: evpiva@gmail.com

**Keywords:** pyrazoles, *Candida albicans*, antifungal activity, anti-enzyme activity

## Abstract

Because of the need for more effective and less harmful antifungal therapies, and interest in the synthesis of new carboximidamides, the goal of this study was to determine the antifungal and anti-enzyme activities of some new pyrazole carboximidamides and their cytotoxicity. For this purpose, tests were performed to evaluate: minimum inhibitory concentration (MIC) and minimum fungicidal concentration (MFC); production of proteinases and phospholipase, and cytotoxicity of the extracts. Data were analyzed by ANOVA and Tukey Tests (α = 5%). The results were: MIC and MFC ≥ 62.5 μg/mL (*C. albicans*, *C. parapsilosis*, *C. famata*, *C. glabrata*, and *Rhodotorula mucillaginosa*) and MIC and MFC ≥ 15.6 μg/mL (*C. lipolytica*). The values of proteinase and phospholipase (Pz) of *C. albicans* before and after exposure to the compounds were: 0.6 (±0.024) and 0.2 (±0.022) and 0.9 (±0.074) and 0.3 (±0.04), respectively. These proteinase results were not significant (*p* = 0.69), but those of phospholipase were (*p* = 0.01), and 15.6 μg/mL was the most effective concentration. The cytotoxicity means were similar among the tests (*p* = 0.32). These compounds could be useful as templates for further development through modification or derivatization to design more potent antifungal agents. Data from this study provide evidence that these new pyrazole formulations could be an alternative source for the treatment of fungal infections caused by *Candida*. However, a specific study on the safety and efficacy of these *in vivo* and clinical trials is still needed, in order to evaluate the practical relevance of the *in vitro* results.

## 1. Introduction

*Candida* species are important pathogens correlated with 8% to 10% of the opportunistic infections capable of affecting the skin, mouth, esophagus, gastrointestinal tract, vagina and vascular system [1]. These infections have increased over the past several years and represent a challenge, especially to immunocompromised patients [2]. *C. albicans* is regarded as being the main causative agent of these infections, which are often related to predisposing local and systemic factors. This species has become a more pathogenic and tough genre because of its ability to coalesce and form heterogeneous biofilms, and irrespective of the mechanism involved, may have an impact on the selection of antimicrobial therapy and the response to infections associated with antimicrobial therapy [3]. However, other *Candida* species have been correlated with increased fungal infections, such as *C. tropicalis*, *C. parapsilosis*, *C. stellatoidea*, *C. glabrata* and *C. krusei* [4]. Some evidence indicates that increased use of fluconazole or other antifungal agents may be responsible for fungal infections (other than *C. albicans*) with reduced antifungal susceptibility [5].

Even with this important overview of fungal infections and increase in the number of antifungal agents on the market, there is still a wide gap in the effectiveness of treatment of these infections, in comparison with the results obtained with antibacterial therapy [2]. Furthermore, the biochemical and physiological similarities between fungal and human cells provide many substances with deleterious actions and nonspecific antifungal properties, which can causing a number of side effects during therapy [6]. Furthermore there is also concern about the increasing pathogenicity of the less virulent *Candida albicans* species [7]. Considering the aspects of diagnosis and therapy, these infections are a significant challenge to medicine [8]. Thus, there is a need to encourage research and innovative efforts with regard to new antifungal compounds.

Heterocyclic compounds containing the pyrazole unit have a broad spectrum of biological activities, such as monoamine oxidase inhibitor [9], anticonvulsant [10], antibacterial [11], hypotensive [12], antipyretic [13] and anti-inflammatory [14] activity. In addition, 1-carboxamidino has replaced pyrazole, and its derivatives have been used for the synthesis of several bi-heterocycles [13] and as amidinating reagents to convert primary and secondary amines into the corresponding guanidines [15].

There were recent reports of the regiospecific synthesis of many pyrazole derivatives under mild conditions via (3+2) cyclocondensations between 1,3-dielectrophiles and hydrazines [16,17,18]. In addition, there have been reports of the synthesis of a series of 5-aryl-1-carboxamidino-3-styryl-4,5-dihydro-1*H*-pyrazole derivatives from the cyclocondensation of 1,5-diaryl-1,4-pentadien-3-ones and aminoguanidine hydrochloride and their application in the synthesis of new 2-(5-aryl-3-styryl-4,5-dihydro-1*H-*pyrazol-1-yl)-4-(trifluoromethyl)pyrimidines [19]. Furthermore, the synthesis under mild conditions of novel 3,5-diaryl-4,5-dihydro-1*H*-pyrazole-1-carboximidamides by the reaction of chalcones with aminoguanidine hydrochloride in KOH has been described [20]. In addition to the antioxidant capacity of pyrazoles [16,17] several potential applications should be investigated.

Because of the need for more effective and less harmful antifungal therapies, and the availability of novel syntheses of new carboximidamides, the goal of this study was to determine the antifungal and anti-enzyme activities of some new pyrazole carboximidamides and their cytotoxicity. The hypothesis to be tested was that 3,5-diaryl-4,5-dihydro-1*H*-pyrazole-1-carboximidamides would show antifungal activity against *Candida* and low cytotoxicity.

## 2. Results and Discussion

### 2.1. Chemistry

**Scheme 1:**
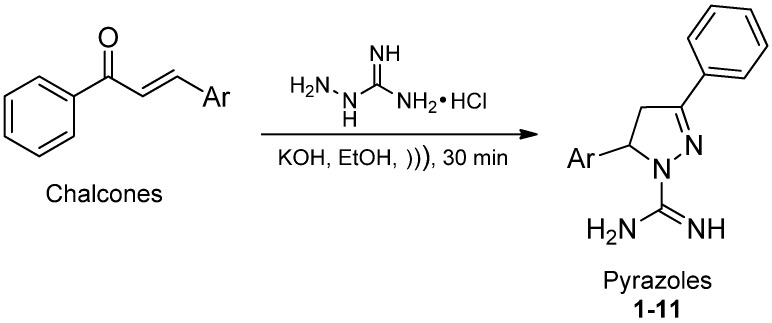
General synthesis of the 3,5-diaryl-4,5-dihydro-1*H*-pyrazole-1-carboximidamides **1**–**11** [18].

The tested pyrazoles **1**–**11** were obtained by the reaction of chalcones with aminoguanidine hydrochloride in ethanol under ultrasound irradiation (Scheme 1).

**Figure 1:**
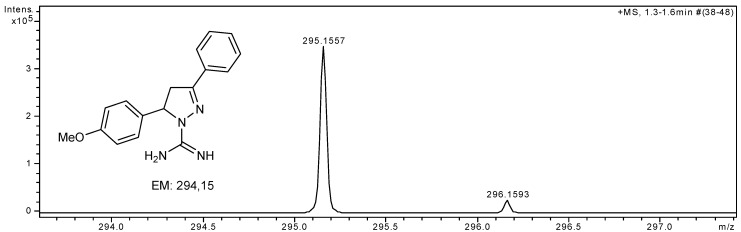
Representative HRMS spectrum of 5-(4-methoxyphenyl)-3-phenyl-4,5-dihydro-1*H*-pyrazole-1-carboximidamide (**1**).

The preparation, yields and the ^1^H- and ^13^C-nuclear magnetic resonance (NMR) data of compounds **1**–**11** are reported in the literature [18,20]. In this work, the structures of compounds **1**–**11** were confirmed by High Resolution Mass Spectrometry (HRMS). The representative spectrum of compound **1** is shown in Figure 1 and selected experimental High Resolution Mass Spectrometry data for compounds **1**–**11** are given in Table 1.

**Table 1:** Experimental High Resolution Mass Spectrometry data for compounds **1**–**11**.

Pyrazole ^a^	Molecular Formula	HRMS *m/z* [MH]^+^	
		Calculated	Found
**1**	C_17_H_19_N_4_O	295.1559	295.1557
**2**	C_16_H_17_N_4_	265.1453	265.1452
**3**	C_16_H_16_ClN_4_	299.1063	299.1047
**4**	C_16_H_16_BrN_4_	343.0558	343.0547
**5**	C_17_H_19_N_4_O	295.1559	295.1550
**6**	C_16_H_15_Cl_2_N_4_	333.0674	333.0685
**7**	C_17_H_19_N_4_	279.1610	279.1610
**8**	C_18_H_21_N_4_O_2_	325.1664	325.1661
**9**	C_16_H_16_N_5_O_2_	310.1304	310.1318
**10**	C_16_H_16_BrN_4_	343.0558	343.0546
**11**	C_17_H_19_N_4_	279.1610	279.1602

^a^ (**1**) 5-(4-Methoxyphenyl)-3-phenyl-4,5-dihydro-1*H*-pyrazole-1-carboximidamide; (**2**) 3,5-Diphenyl-4,5-dihydro-1*H*-pyrazole-1-carboximidamide; (**3**) 5-(4-Chlorophenyl)-3-phenyl-4,5-dihydro-1*H*-pyrazole-1-carboximidamide; (**4**) 5-(2-Bromophenyl)-3-phenyl-4,5-dihydro-1*H*-pyrazole-1-carboximidamide; (**5**) 5-(2-Methoxyphenyl)-3-phenyl-4,5-dihydro-1*H*-pyrazole-1-carboximidamide; (**6**) 5-(2,4-Dichlorophenyl)-3-phenyl-4,5-dihydro-1*H*-pyrazole-1-carboximidamide; (**7**) 5-(4-Methylphenyl)-3-phenyl-4,5-dihydro-1*H*-pyrazole-1-carboximidamide; (**8**) 5-(3,4-Dimethoxyphenyl)-3-phenyl-4,5-dihydro-1*H*-pyrazole-1-carboximidamide; (**9**) 5-(3-Nitrophenyl)-3-phenyl-4,5-dihydro-1*H*-pyrazole-1-carboximidamide; (**10**) 5-(4-Bromophenyl)-3-phenyl-4,5-dihydro-1*H*-pyrazole-1-carboximidamide; (**11**) 5-(2-Methylphenyl)-3-phenyl-4,5-dihydro-1*H*-pyrazole-1-carboximidamide.

### 2.2. Biological Activity

The choice of the broth dilution test to determine the sensitivity of the yeasts to antifungal therapy, as recommended by The National Committee for Clinical Laboratory Standards (NCCLS, M27-A2 protocol), was based on the advantages of easy reproducibility [21], low cost and sensitivity. In addition, this method requires only a small amount of each sample, which can be used for various tests [22]. The standard M27-A2 methodology recommends reading the results for *Candida* after 48 h. In our study, we chose to read the results at two time intervals, 24 and 48 h, due to the lack of knowledge about the possible activity and behavior of these novel compounds. The two readouts showed the occurrence of trailing and partial inhibition of growth over a wide range of concentrations of the antifungal agent.

The research hypothesis was confirmed since data from this study provide evidence that these new formulations of pyrazoles could be an alternative source for the treatment of fungal infections caused by *C. albicans* and non- *C. albicans*. The fungal species chosen for this study were *Candida albicans*, *Candida* non*-albicans* (*C.parapsilosis*; *C famata*; *C. glabrata*; *C. lipolytica*) and *Rhodotorula mucillaginosa*. This choice reflects the fact that *Candida* spp. are responsible for the majority of fungal infections [23]. On the other hand, the widespread use of antifungal therapies and prophylaxis has resulted in an increasing number and severity of the infections caused by other non-*albicans* species of *Candida*, mainly *C. glabrata* and *C. parapsilosis* [24], making it is essential to study treatment alternatives for these other *Candida* species as well. Table 2 shows the results for the minimum inhibitory concentration (MIC) and minimum fungicidal concentration (MFC) of the pyrazole derivatives **1**–**11** dissolved in dimethyl sulfoxide (DMSO), or in 70% ethanol, respectively, as assessed against strains of *C. albicans*. For most of the compounds, the MIC and MFC values were similar and indicated that several of the compounds were potential fungicidal agents. Three pyrazole derivatives, compounds **8**, **9** and **11**, failed to exhibit any antifungal activity in this study.

It was shown that most of the pyrazole derivatives had a similar effect at 125–250 μg/mL. However, compound **5** showed a MIC and a MFC of 15.6 μg/mL against one of the strains of *Candida albicans*. This result can be compared with other studies that used the same methodology to test promising novel antifungal compounds, for example, Tamoxifen and Clomiphene [25].

Table 2 shows the results of the MIC and MFC of the pyrazole derivatives assessed against strains of non-C. *albicans* species. In recent times, there has been a greater recognition of the importance of non-*Candida albicans* species in human disease. *C. glabrata* recently received special attention because of its greater resistance to certain antifungal agents, arousing pharmacological interest [26].

However, inhibition of fungal growth alone may not be sufficient to prevent the spread of *Candida* in immunosuppressed patients [27]. Until recently, fungal infections were generally correlated with opportunism in debilitated or otherwise weakened patients. Although debilitated patients comprise a large percentage of those infected by *Candida* spp., it is recognized today that candidiasis may also develop in otherwise healthy individuals [28].

The interaction between yeast and human cells is essential for the colonization and invasion of tissues, and thus, for the onset and establishment of the disease. *C. albicans* uses three strategies for pathogenicity and invasion: (a) escape from the immune responses [29]; (b) changes in the morphogenic shapes of the yeast hyphae, which enhance the ability of yeast to adhere to and invade host cells; and (c) the invasion of host cells, supported by factors associated with the hyphae, such as the secretion of hydrolytic enzymes [30]. Aspartyl proteinases (Saps) and phospholipases are among the major hydrolytic enzymes secreted. The proteases are responsible for degrading various physiologically important substrates [30] and the phospholipases are directly associated with adhesion to the epithelial tissue of the yeast host [31]. Thus, the study of the activity of these enzymes and of substances capable of inhibiting their release is important for the development of new alternatives for the treatment of candidiasis.

**Table 2:** *In vitro* antifungal activity against *Candida albicans* and non- *C.albicans* of the pyrazole derivatives dissolved in DMSO or in 70% ethanol.

Compound	Structural Formula	Chemical Formula	*C. albicans* (*n* = 33) Median (range)		*R. mucillaginosa* (*n* = 2)		*C. parapsilosis* (*n* = 2)		*C. famata* (*n* = 2)		*C. glabrata* (*n* = 2)		*C. lypolytica* (*n* = 2)	
			DMSO	70% ethanol	DMSO	70% ethanol	DMSO	70% ethanol	DMSO	70% ethanol	DMSO	70% ethanol	DMSO	70% ethanol
			MIC µg/mL	MFC µg/mL	MIC µg/mL	MFC µg/mL	MIC µg/mL	MFC µg/mL	MIC µg/mL	MFC µg/mL	MIC µg/mL	MFC µg/mL	MIC µg/mL	MFC µg/mL
**1**	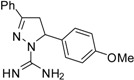	C_17_H_18_N_4_O	250	>250	250	250	>62.5	>62.5	>62.5	>62.5	125	125	15.6	15.6
**2**	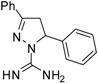	C_16_H_16_N_4_	250	250	125	125	125	125	>62.5	>62.5	62.5	62.5	31.2	31.2
**3**	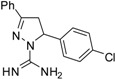	C_16_H_15_ClN_4_	125	125	250	250	62.5	62.5	62.5	62.5	62.5	62.5	31.2	31.2
**4**	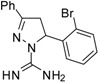	C_16_H_15_BrN_4_	250	250	250	250	125	125	>62.5	>62.5	62.5	62.5	>31.2	>31.3
**5**	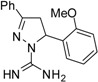	C_17_H_18_N_4_O	15.6	15.6	62.5	62.5	>62.5	>62.5	62.5	62.5	125	125	15.6	15.6
**6**	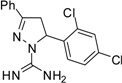	C_16_H_14_Cl_2_N_4_	250	250	250	250	125	125	125	125	125	125	>15.6	>15.6
**7**	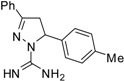	C_17_H_18_N_4_	250	>250	250	250	125	125	125	125	125	125	62.5	62.5
**8**	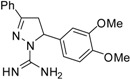	C_18_H_20_N_4_O_2_	-	-	-	-	-	-	-	-	-	-	-	-
**9**	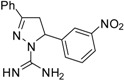	C_16_H_15_N_5_O_2_	-	-	-	-	-	-	-	-	-	-	-	-
**10**	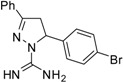	C_16_H_15_BrN_4_	125	125	250	250	125	125	125	125	>125	>125	<62.5	<62.5
**11**	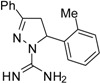	C_17_H_18_N_4_	-	-	-	-	-	-	-	-	-	-	-	-

No differences were observed in the MIC and MFC values for compounds dissolved in DMSO or 70% ethanol. Thus, it is preferable for the compounds derived from pyrazoles with antifungal activity *in vitro* to be dissolved in 70% ethanol, which is less toxic and is compatible with pharmaceutical preparations for the treatment of candidiasis.

The precipitation zone (Pz) values for proteinase and phospholipase of *C. albicans* before and after exposure to the compounds were: 0.2 (±0.022) and 0.6 (±0.024) and 0.3 (±0.04) and 0.9 (±0.074), respectively. These proteinase results were not significant. The phospholipase results were (*p* = 0.01) and 15.6 mg/mL was the most effective concentration. For both tests, the addition of 3,5-diaryl-4,5-dihydro-1*H*-pyrazole-1-carboximidamides resulted in decreased secretion of phospholipase and proteinase in the strains tested (*p* < 0.001—Spearman Correlation). Other studies have tested compounds capable of inhibiting phospholipase, such as chlorhexidine [32]. Clearly there is a need for further studies in order to clarify the mechanism of action of the new substances tested here, in order to eventually establish them as a feasible alternative treatment for the various diseases caused by *Candida* spp.

**Figure 2:**
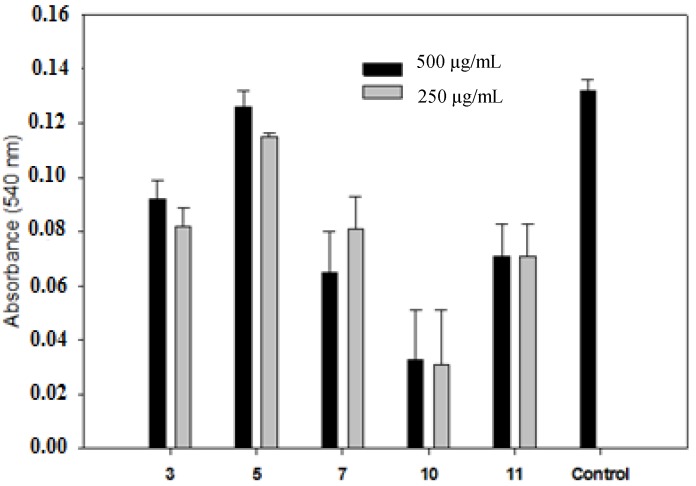
Cytotoxicity of compounds dissolved in DMSO after 24 h of cell exposure. Compounds showed statistically similar cytotoxicity levels.

With regard to cytotoxicity, the present findings showed that the cytotoxic effect of compounds were not significantly different from those obtained for the control group (Figure 2), reinforcing the potential for therapeutic use of these antifungal agents. Among antifungal agents, the therapeutic agents most frequently used for the topical treatment of oral candidiasis are polyenes (nystatin and amphotericin B) and azoles (itraconazole, miconazole and clotrimazole) [33,34]. Although they are widely used, they have certain limitations due to side effects such as toxicity and the emergence of resistant strains [35], thus reaffirming the need for the development of other compounds with low cytotoxicity and the potential for treatment of mycoses [36].

In view of the difficulties encountered in the treatment of fungal infections, some authors have recommended the isolation of the causative agent of infection, and determination of the minimum inhibitory concentration and minimum fungicidal concentration of the drugs that might potentially be used. A minimum inhibitory concentration of the chosen antimicrobial should be established by a standardized laboratory method to ensure susceptibility [37]. However, establishing the antifungal activity of a new drug *in vitro* provides only part of the information needed to predict the outcome of treatment with a new drug. This reflects the fact that the interaction between antifungal drugs and fungi, the fungus and the host, and the host and the antifungal drugs is very complex. Nonetheless, at present, *in vitro* assays and susceptibility tests still continue to be indispensable tools for selecting the most appropriate antifungal agent [38]. In particular, protocols for monitoring patients with Acquired Immunodeficiency Syndrome (AIDS) or other immunocompromising diseases, who have an increased frequency of fungal infections, should routinely be included in a rapid laboratory confirmation of the presence of fungal species and their susceptibility profile. New antifungal drugs for the treatment of oral candidiasis [39] should be a particular area of interest to the pharmaceutical industry.

The remarkable antifungal effects of some of the pyrazole compounds assessed in this study could be a useful lead for the development of new and promising antifungal agents against oral candidiasis, mainly caused by *C. albicans*. However, further pharmacological and toxicological studies will be necessary to confirm this hypothesis.

Compound **5**, bearing a methoxy group at the *ortho* position, was the most active of all of the compounds tested. It is the only one that has a hydrogen bond acceptor group at this position, suggesting that this feature might be decisive for its greater activity. Compounds **4** and **10** have similar oil-water partition coefficients, but different activities, indicating that although it promotes greater cell penetration, lipophilicity is not the determining factor for activity. These assumptions are reinforced by a comparison of the MIC of compounds **3** and **6**, for which an inverse relationship between biological activity and lipophilicity was observed. Analogs **3** and **6** and **4** and **10** have similar hydrophobicities and show that the presence of a halogen atom at the *ortho* position decreases the activity. Compounds **5** and **1** provide additional evidence for the importance of the substituent position on the aromatic ring, where the presence of the methoxy group at the *para* position greatly reduces the activity. Derivatives bearing the *meta* substituent were inactive. This study underlines the importance of the *ortho* position for activity and certainly will guide us in the design of the next generation of antifungal compounds. In the future work, we will be testing compounds with different groups at the *ortho* position with distinct steric, electronic, hydrophobic or hydrophilic features and evaluate the impact of these structural variations on the antifungal activity.

The largest class of antimycotics available today, the azoles, act by inhibiting ergosterol biosynthesis through inhibition of 14-*α*-demethylase, a key heme enzyme in the biosynthetic pathway. This inhibition results in the accumulation in the fungal cell membrane of anomalous steroids with different shapes and physical properties from the normal membrane sterol ergosterol. The effects arising from this modification lead to fungal cell death. The N-3 atom of the heterocyclic aromatic ring of azoles (imidazole or triazole) is essential for the activity of these compounds. Based on this fact, our further research will verify whether aromatization of the heterocyclic ring increases the anti-*Candida* activity.

## 3. Experimental

### 3.1. General Information

The 3,5-diaryl-4,5-dihydro-1*H*-pyrazole-1-carboximidamides **1**–**11** were prepared according to the literature and characterized by ^1^H, ^13^C-NMR [18,20], and confirmed by HRMS. All solvents and reagents were obtained from Aldrich (Hamburg, Germany) and used without further purification. NMR spectra were recorded on a Bruker DPX 300 spectrometer (Bremen, Germany) at 300.13 MHz for ^1^H and 75.48 MHz for ^13^C) at 300 K. The progress of reactions was monitored on a Thermo Trace GC Ultra chromatograph (Ramsey, MN, USA) equipped with a Varian FactorFour™ capillary column (95% dimethyl- and 5% diphenylpolysiloxane; 0.25 mm × 30 m; column head pressure, 14 psi). The mass spectra were obtained on an Applied Biosystems LC/MS/MS Hybrid Triple Quadrupole Linear Ion Trap System, Model 3200 QTRAP (Cole-Parmer, Vernon Hills, IL, USA). The parameters were: Curtain Gas (CUR) = 10, Ion Spray Voltage (IS) = 5500, Temperature (TEM) = 25 °C, Ion Source Gas (GS1) = 15, Ion Source Gas (GS2) = 0, Declustering Potential (DP) = 70, Entrance Potential (EP) = 10 and Scan and Product Ion (MS2). The reactions were carried out with a microtip probe connected to a 500 W Sonics Vibra-cell ultrasonic processor (Newtown, Connecticut, USA) operating at 20 KHz with 25% of the maximum power output.

### 3.2. Strains

*C. albicans* (33), *C. parapsilosis* (2), *C. famata* (2), *C. glabrata* (2), *C. lipolytica* (2) and *R. mucillaginosa* (2) strains were used for determining the *in vitro* anti-*Candida* activity of eleven 3,5-diaryl-4,5-dihydro-1*H*-pyrazole-1-carboximidamides. These strains of *C. albicans* were collected from denture user patients with Chronic Atrophic Candidiasis (CAC), enrolled in the Center for Diagnosis of Diseases of the Mouth (CDDB), School of Dentistry, Federal University of Pelotas (FOP-UFPel). The diagnosis of CAC was clinical and microbiological, as previously described [40].

### 3.3. In Vitro Antifungal Activity

#### 3.3.1. Inoculum

The strains of *C. albicans* and non- *C. albicans* were sub-cultured on Sabouraud Dextrose Agar with 0.2 mg/mL of chloramphenicol at 36 °C for 24 h. After incubation, these strains were individually inoculated into tubes containing 5 mL of a sterile 0.85% saline solution and the yeast suspension was adjusted to a 0.5 McFarland standard (which is approximately 10^6^ colony forming units CFU/mL). The inoculum was then resuspended to obtain a final concentration in Roswell Park Memorial Institute (RPMI) 1640 medium (Sigma, St. Louis, MO, USA) buffered to pH 7.0 with 165 mmol L^−1^ of morpholinepropanesulfonic acid (MOPS; Vetec, Duque de Caxias, Rio de Janeiro, Brazil).

#### 3.3.2. Determination of the MIC and MFC

MICs and MFCs of the new pyrazole derivatives were determined by using the broth microdilution techniques described by the Clinical and Laboratory Standards Institute for yeasts (CLSI, M27-A2 protocol). Stock solutions of these eleven new pyrazole derivatives were prepared in DMSO and in 70% ethanol solution at the concentration of 500 μg/mL. The solutions were diluted in Roswell Park Memorial Institute (RPMI) 1640 medium (Sigma) and the final drug concentrations ranged from 0.49 to 500 μg/mL. Two replicates were performed for each concentration of the compounds tested against the strains of *Candida albicans* and non- *C. albicans*.

For the test, 20 µL of each concentration of each compound were collected, and 1980 µL of Roswell Park Memorial Institute (RPMI) 1640 medium (Sigma) were added. For the test, 20 µL of each concentration of each compound were collected, and 1980 µL of Roswell Park Memorial Institute (RPMI) 1640 medium (Sigma) were added. After preparation and dissolution of the above-mentioned compounds, 100 mL of each compound were pipetted into each well of the test plate, to which another 100 mL of the innoculum were added. For the positive control test plate, we added 100 mL of the innoculum and did not include the compounds to be tested. For the negative control, we added 100 mL of the compound and did not add the innoculum.

After 48 h of incubation at 35 °C, the MIC was determined visually by comparison with the drug-free growth control well. Each inoculum from the previous test that did not show growth was subcultured on agar plates. After 24 h of incubation, the readout was determined by the visible growth of strains. The MFC was considered the lowest concentration that prevented visible growth.

### 3.4. In Vitro Anti-Enzyme Activity

#### 3.4.1. Phospholipase

To conduct the tests, all samples were cultured on Sabouraud Dextrose Agar 4% for 24 h. After this period, samples were diluted in sterile distilled water at a 0.5 McFarland scale. After preparing the dilution, 20 mL of each sample was placed in the test medium. Reduced egg yolk agar (Reya) was used and the plates were inoculated (four per isolate) with a 5 μL of inoculum suspension was plated on equidistant points of the surface of Reduced egg yolk agar (Reya) and left to dry at room temperature. The plates were then incubated aerobically at 37 °C for 48 h and the diameters of the precipitation zones around the colonies were measured. The enzyme activity was expressed as the Precipitation Zone (Pz) value, determined from the diameter of colony/(diameter of the hyaline zone) and scaled as: (Pz = 1, Negative; 1 > Pz ≥ 0.64, Semi-positive; Pz ≤ 0.63, Positive). Each experiment was repeated twice [32].

#### 3.4.2. Proteinase

To conduct the tests, all samples were cultured on Sabouraud Dextrose Agar 4% for 24 h. After this period, samples were diluted in sterile distilled water at a 0.5 McFarland scale. After preparing the dilution, 20 mL of each sample was placed in the test medium. Clinical isolates were inoculated in tubes containing 5 mL of Yeast Extract-Peptone-Dextrose (YPD) medium and incubated at 37 °C for 18 h. After the incubation, 1.5 mL aliquots of the culture were transferred to Eppendorf tubes at 4 °C and centrifuged at 3000 rpm for 5 min. The cell pellets were resuspended in saline solution (0.9% NaCl) and centrifuged twice more under the same conditions to remove cultivation debris. The concentrations of the suspensions of the strains were standardized by using the index range of 0.5 MacFarland (1 × 10^6^ CFU/mL) and 20 µL volumes were inoculated at equidistant points in the middle of Proteinase Agar plates. The plates with different inocula were incubated at 37 °C for four days. The tests were performed in duplicate. The Precipitation Zone (Pz) values were determined from the radius of the diameter of colony/hyaline zone (Pz = 1, Negative; 1 > Pz ≥ 0.64, Semi-positive; Pz ≤ 0.63, Positive.) [33].

### 3.5. Cytotoxicity Assay

The cell culture medium was Dulbecco’s Modified Eagle’s Medium (DMEM) supplemented with 10% fetal bovine serum (FBS), 2% l-glutamine, penicillin (100 U/mL) and streptomycin (100 mg/mL). Mouse fibroblasts of the 3T3/NIH immortalized cell line were maintained as a stock culture in DMEM and incubated at 37 °C in a humidified atmosphere of 5% CO_2_ in air until subconfluency.

The 3-(4,5-dimethylthiazol-2-yl)-2,5-diphenyltetrazolium bromide (MTT) assay was used to assess cell metabolic function according to mitochondrial dehydrogenase activity. Mouse fibroblasts (3T3/NIH; 2 × 10^4^/cells) were maintained in DMEM in 96 well plates for 24 h. DMEM was removed and replaced with 200 µL of extract from different groups with 10% FBS. Cytotoxicity produced by the different extracts was assessed at 24 h of cell exposure time. After removing the extracts, the cells were washed with phosphate-buffered saline (PBS) and then 200 µL of medium in 20 µL of MTT solution (5 mg of MTT/mL DMEM) were added to each well. After 5 h of incubation at 37 °C in the dark, the blue formazan precipitate was extracted from the mitochondria using 200 µL/well of DMSO on a shaker at 150 rpm for 5 min. The absorption at 540 nm was determined spectrophotometrically. [41]

The cell viability was analyzed using SigmaStat 3.5 (Systat Software Inc., San Jose, CA, USA) statistical analysis software program. The statistical analysis was performed by one-way ANOVA with the level of significance set at *p* < 0.05.

## 4. Conclusions

3,5-Diaryl-4,5-dihydro-1*H*-pyrazole-1-carboximidamides could be useful as templates for further development through modification or derivatization to design more potent antifungal agents. Data from this study provide evidence that these new formulations of pyrazoles could be an alternative source for the treatment of fungal infections caused by *Candida*. It was clear that many of the synthesized compounds exhibited good antifungal activity for *C. albicans* and low cytotoxicity. However, a specific study on the safety and efficacy of these *in vivo* and clinical trials is still needed to evaluate the practical relevance of *in vitro* results.
